# Phytochemical analysis and antioxidant activity of *Lycium barbarum* (Goji) cultivated in Greece

**DOI:** 10.1080/13880209.2016.1265987

**Published:** 2016-12-09

**Authors:** Amina Benchennouf, Spyros Grigorakis, Sofia Loupassaki, Eugene Kokkalou

**Affiliations:** a Department of Food Quality and Chemistry of Natural Products, Mediterranean Agronomic Institute of Chania (MAICh), Chania, Greece;; b Laboratory of Pharmacognosy-Phytochemistry, Department of Pharmacognosy-Pharmacology, School of Pharmacy, Aristotle University of Thessaloniki, Thessaloniki, Greece

**Keywords:** Goji berry, polyphenols, chemiluminescence, LC-DAD-MS (ESI+)

## Abstract

**Context:** The fruit of *Lycium barbarum* L. (Solanaceae), known as goji berry, has been exploited for a long time in traditional Chinese medicine. In recent decades, it has received much attention as one of the trendiest functional foods with a wide array of pharmacological activities in Western diets.

**Objective:** In this study the phenolic profile and potential antioxidant capacity of *Lycium barbarum* cultivated in Crete (Greece) were investigated.

**Materials and methods:** The berries were defatted with hexane and then extracted with dichloromethane and methanol using a Soxhlet apparatus. Furthermore, the methanol extract was fractionated with ethyl acetate and butanol. All fractions/extracts were tested for their antioxidant activity (DPPH, FRAP, chemiluminescence). Folin–Ciocalteu and LC-DAD-MS analyses were utilized for the identification of the phenolic compounds.

**Results:** The total phenolic content ranged from 14.13 ± 0.40 (water fraction) to 109.72 ± 4.09 (ethyl acetate fraction) mg gallic acid equivalent/g dry extract. Ethyl acetate extract exhibited the highest scavenging activities determined as EC_50_ (4.73 ± 0.20 mg/mL) and IC_50_ (0.47 ± 0.001 mg/mL) using DPPH and chemiluminescence assays. Seventeen phenolic compounds, including cinnamoylquinic acids and derivatives, hydrocinnamic acids and flavonoid derivatives, were tentatively identified. To the best of our knowledge, quercetin 3-*O*-hexose coumaric ester and quercetin 3-*O*-hexose-*O*-hexose-*O*-rhamnose are reported for the first time in goji berry fruits.

**Discussion and conclusion**: The results of this study suggest that consumption of goji berry fruits could serve as a potential source of natural antioxidant compounds and that goji berry phenolic extracts could be exploited for nutritional pharmaceutical purposes.

## Introduction

Increasing evidence for a relationship between diet and health highlight the importance of plant secondary metabolites and their impact on different physiological functions and health (Verschuren 2002). This knowledge has generated new concepts in nutrition aiming at developing and promoting functional foods, known to be rich with such bioactive compounds (Roberfroid 2002). Goji berry [*Lycium barbarum* L. (Solanaceae)], also known as wolfberry, can be considered one of those foods (Carnés et al. 2013); pharmacological and immunological studies seem to support some of the claims made with regard to their health-promoting properties (Gan et al. 2004; Song et al. 2011).

Since the beginning of the twenty-first century, goji berries became increasingly popular in Europe and North America (Istrati et al. 2013) given its nutritional richness in various vitamins, minerals, antioxidants, and amino acids (Yao et al. 2011; Endes et al. 2015). This might explain the rapid increase in consumption in recent years.

In parallel to all these nutritional benefits, goji fruit might confer many health-protective benefits such as age-related macular degeneration, which can be due to the presence of lutein and zeaxanthin (Bucheli et al. 2011). In addition, it may possess antioxidant and antitumor activities (Gan et al. 2004; Zhang et al. 2010), neuroprotective effects (Lo & Yang 2015), male fertility facilitation (Lau et al. 2012) as well as immunity enhancement (Zhang et al. 2015). However, the complete profile with quality traits, phytochemical composition and antioxidant activity evaluation of these berries is still lacking.

Therefore, the present study aimed at characterizing the phenolic profile, and to evaluate the potential antioxidant activity of goji berries, cultivated on the island of Crete (Greece). The antioxidant activity was evaluated using DPPH scavenging activity, ferric-reducing antioxidant power (FRAP), and luminol-induced chemiluminescence methods. The Folin–Ciocalteu method was used to determine the total phenolic content of each one of the extracts/fractions while the polyphenolic profile was determined using liquid chromatography-diode array-mass spectrometry (LC-DAD-MS). The extracts/fractions examined were prepared using successive solvents of varying polarity and by partitioning the methanol extract with ethyl acetate and butanol.

## Materials and methods

### Plant material

Fully ripened goji fruits (*Lycium barbarum)* were picked from an organic farm located in Chania (Crete, Greece) in September 2014. The plant was identified and authenticated by Dr. Theano Samara and a voucher specimen (EK 62) was deposited at the laboratory of ‘Food Quality and Chemistry of Natural Products’ (MAICh, Greece). The berries were left to dry in an oven at 40 °C for 48 h. The obtained dry material was ground using an electric blender, weighed and subsequently subjected to further processes.

### Chemicals and reagents

Acetic acid, ethyl acetate, cobalt (II)-chloride hexahydrate, Folin–Ciocalteau reagent, gallic acid, glacial acetic acid (C_2_H_4_O_2_), hydrochloric acid and hydrogen peroxide (H_2_O_2_, 30%) were purchased from Merck (Germany). Boric acid (H_3_BO_3_), methanol, butanol, 2,2-diphenyl-1-picrylhydrazyl (DPPH), EDTA (ethylenediamine tetraacetic acid), luminol (3-aminophtalylhydrazide), 2, 4, 6-tripyridyl-s-triazine (TPTZ), iron (III) chloride hexahydrate (FeCl_3_·6H_2_O), iron (II) sulfate heptahydrate (FeSO_4_·7H_2_O), sodium acetate trihydrate (C_2_H_3_NO_2_), sodium carbonate, and sodium hydroxide were purchased from Sigma-Aldrich (Germany).

### Extraction and partitioning of extracts

Approximately 20 g of ground fruits were subjected to extraction using a Soxhlet apparatus, with the following solvents in order of increasing polarity: hexane, dichloromethane, and methanol. The hexane extract was discarded while the dichloromethane and methanol extracts were evaporated under vacuum with a rotary evaporator. The methanol extracts were dissolved in 300 mL of boiling water and then fractionated three times sequentially with ethyl acetate and butanol. These three fractions were each concentrated as described above.

### Estimation of the total phenolic content by the Folin–Ciocalteau test

**Figure 1.**
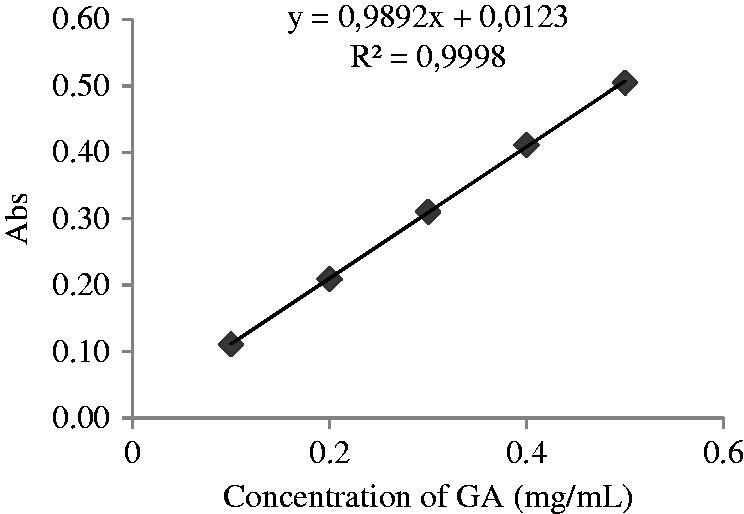
Calibration curve (gallic acid).

The amount of total phenolics in fruit extracts was measured following the Folin–Ciocalteu procedure (Arnous et al. 2002). In a 1.5 mL Eppendorf tube, 790 μL of distilled water, 10 μL of diluted sample and 50 μL of Folin–Ciocalteau reagent were added and the mixture vortexed. After 1 min, 150 μL of aqueous sodium carbonate (20%) was added and the mixture vortexed and allowed to stand at room temperature without light for 120 min. The absorbance was read at 750 nm. The total polyphenol concentration was calculated from a calibration curve (Figure 1) (100–500 μg/mL) and the results were expressed as mg of gallic acid equivalents (GAE)/g dry extract.

### Antioxidant activity determinations

#### Evaluation of the antioxidant activity using the DPPH^•^ method

The DPPH assay was performed according to the method described by Arnous et al. (2002). Briefly, a methanol DPPH solution (0.1 mM, 975 μL) was added to 25 μL of different concentrations of extracts/fractions. The mixture was shaken vigorously and the decrease in absorbance was measured at 515 nm after 30 min of incubation in the dark with methanol as the blank. The percentage of DPPH inhibition was calculated as follows:

$$\%\;\mathrm{DPPH}=\left[\frac{A_{\mathrm{DPPH}}-A_{S}}{A_{\mathrm{DPPH}}}\right]\times 100$$

where A_S_ is the absorbance of the solution when the sample extract has been added at a particular level and A_DPPH_ is the absorbance of the DPPH solution. The half maximal effective concentration (EC_50_) value (mg/mL) is the effective concentration at which the DPPH radical was scavenged by 50% and calculated by interpolation from the data.

### The Co (II)/EDTA-induced luminol chemiluminescence method

Co (II)/EDTA-induced luminol chemiluminescence measurements were performed as described earlier (Parejo et al. 2000) with some minor modifications. The chemiluminescence (CL) test consisted of mixing 1 mL of a buffer solution of boric acid (50 mM, pH =9) containing CoCl_2_·6H_2_O (2 mg/mL) and EDTA (10 mg/mL) with 100 μL of luminol solution (0.56 mM) in borate buffer (pH =9). Three different dilutions of each sample were prepared. Thereafter, 25 μL of the diluted sample were added and the mixture was vortexed for 15 s. Then, 25 μL of H_2_O_2_ aqueous solution (5.4 mM) was added. The instantaneous reduction in the plateau CL intensity, induced by the addition of sample, was recorded as I, and CL intensity, induced in the absence of the sample, was recorded as I_0_. The ratio (I_0_/I) was plotted vs. the concentration (mg/mL) sample. The equation was established by linear regression and the concentration of sample, which is required to decrease the CL intensity by 50%, was calculated (IC_50_). The hydroxyl free radical scavenging activity (SA_HFR_) was expressed as 1/IC_50_.

### FRAP assay

The FRAP assay was carried out according to Benzie and Strain (1999) with slight modifications. The FRAP reagent was prepared from acetate buffer (pH 3.6), 10 mmol TPTZ (2, 4, 6-tripyridyl-s-triazine) solution in 40 mmol HCl and 20 mmol FeCl_3_·6H_2_O (ferric chloride solution) in proportions of 10:1:1 (v/v), respectively. The FRAP reagent was prepared fresh daily and was warmed to 37 °C in a water bath prior to use. One-hundred microliters of sample was added to 3 mL of FRAP reagent. The reaction mixture was incubated for 4 min at room temperature. The absorbance of the reaction mixture was measured at 593 nm. The standard curve was obtained using FeSO_4_·7H_2_O (concentration range: 100–1000 μmol·L^−1^). The results were expressed in millimoles of Fe^2+ ^equivalents per g of dry extract.

### Statistics

All the measurements were means of triplicate measurements (*n* = 3), including standard deviation (± SD). Data were performed by Microsoft Excel 2010 (Redmond, WA). 

### Survey of the phenolic profile by LC-DAD-MS (ESI+)

Methanol extract fractions with the highest antioxidant capacity (ethyl acetate and butanol fractions) were further screened by LC-DAD/MS (ESI+). The LC-DAD-MS system used was a Finnigan MAT Spectra System P4000 pump, in conjunction with a Finnigan Spectra System UV 6000LP diode array detector and a Finnigan AQA mass analyzer (San Jose, CA). The separation was performed on a Superspher, 100 RP-18,4u end-capped (125 × 2.0 mm) (phenomenex) column with a flow rate was 0.3 mL/min and kept at 40 °C. 

The mobile phase was a linear gradient system of 2.0% acetic acid in water (v/v) (solvent A) and methanol (solvent B). The elution was achieved with the following stepwise gradient: 2 min, 100% A; 10 min, 75% A; 25 min, 75% A; 50 min, 0% A; 60 min, 0% A; 65 min, 100% A. Mass spectra were obtained at the positive ion mode, with acquisition set at 20 and 80 eV, capillary voltage 4.00 kV, source voltage 25 V, detector voltage 650 V and probe temperature 350 °C.

## Results and discussion

### Phenolic content

**Figure 2.**
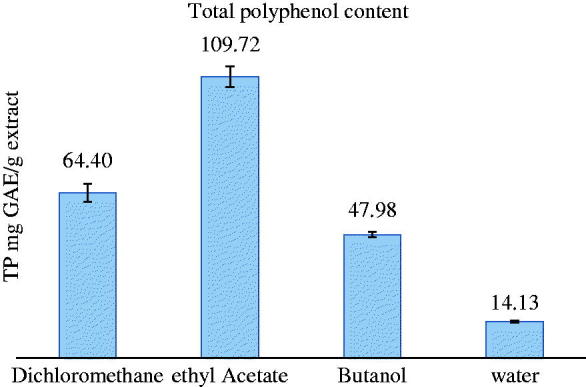
Total polyphenol variation (GAE) of the organic extracts.

The total phenolic content values of goji berry fruit extracts ranged from 14.13 to 109.72 mg GAE/g dry extract (Figure 2). Results were classified according to the solvents that were used for extraction. Ethyl acetate seems to be the solvent that best concentrates the phenolic substances of intermediate polarity. This is in line with previous findings (Termentzi et al. 2006).

### Antioxidant activity assays

Since the antioxidant capacity of food is determined by a mixture of different antioxidants with different mechanisms of action, among which are included synergistic interactions, it is necessary to combine more than one method in order to determine *in vitro* the antioxidant capacity of food stuffs. Therefore, it is necessary to perform more than one type of antioxidant capacity measurement to take into account the various mechanisms of antioxidant action (Frankel & Meyer 2000; Pérez-Jiménez et al. 2008). In this study, the antioxidant activity of the phenolic extracts/fractions isolated from *L. barbarum* fruits were analyzed using DPPH^•^, luminol-induced chemiluminescence and FRAP assays. Table 1 shows the results of the antioxidant activity measured by the three assays.

**Table 1.** Antioxidant activity of the extracts/fractions of the fruit of *Lycium barbarum*.

	DPPH		Chemiluminescence		FRAP
Sample	EC_50_^a^	AE^b^	IC_50_^c^	SA_HFR_^d^	FRAP value^e^
Dichloromethane extract	8.41 ± 0.17	0.12 ± 0.01	0,97 ± 0.01	1.03 ± 0.01	1.20 ± 0.02
Methanolic extract					
Ethyl acetate fraction	4.73 ± 0.20	0.20 ± 0.01	0.47 ± 0.00	2.12 ± 0.01	1.62 ± 0.15
Butanol fraction	12.32 ± 0.11	0.08 ± 0.01	2.27 ± 0.05	0.44 ± 0.01	0.29 ± 0.01
Water fraction	42.76 ± 0.25	0.02 ± 0.00	7.57 ± 0.12	0.13 ± 0.00	0.08 ± 0.01

Results are ± SD (*n* = 3).

a Efficient concentration (mg/mL): The concentration of the sample at which the inhibition rate reaches 50%.

b Antiradical activity: 1/EC_50_.

c Efficient concentration (mg/mL): Concentration of the sample needed to diminish by 50% the initial light emission.

d SA_HFR_: Hydroxyl-free radical scavenging activity (1/IC_50_).

e FRAP value (mmol Fe^2+^/g extract).

According to the DPPH assay in which values are expressed as EC_50_, the ethyl acetate fraction exhibited the highest scavenging activity, followed by the dichloromethane extract, butanol fraction and water fraction, which had the lowest value. This result confirms that most antioxidants were partitioned into the methanol extract. The hydroxyl radical scavenging efficiency, as evidenced from the IC_50_ values obtained with the luminol chemiluminescence test, was found to vary from 0.47 to 7.70 mg dry extract/mL. The SA_HFR_ showed the same pattern and the ethyl acetate fraction had the higher values, while water had the lowest.

As displayed in Table 1, the reduction potential of the iron by the extracts increased in the following order: water fraction < butanol fraction < dichloromethane extract < ethyl acetate fraction. The ability to reduce the FeIII-TPTZ complex can be attributed to the donation of electrons/hydrogen, the mechanism of action that is related to the antioxidant activity of phenolic compounds, which indicates the presence of reducing agents. The ethyl acetate expressed a higher capacity to reduce Fe^3 +^ to Fe^2+^, with a value of 1.62 mmol Fe^2+^/g extract, which may be related to the increased concentration of phenolics in this fraction.

#### Qualitative analysis of phenolics and flavonoid compounds by HPLC-DAD-MS (ESI+)

**Figure 3.**
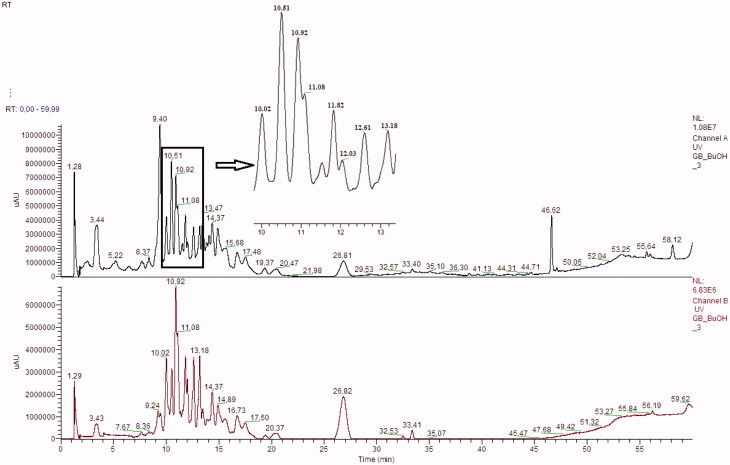
HPLC chromatogram of the ethyl acetate fraction of *L. barbarum* at 278 nm and 340 nm, respectively.

**Figure 4.**
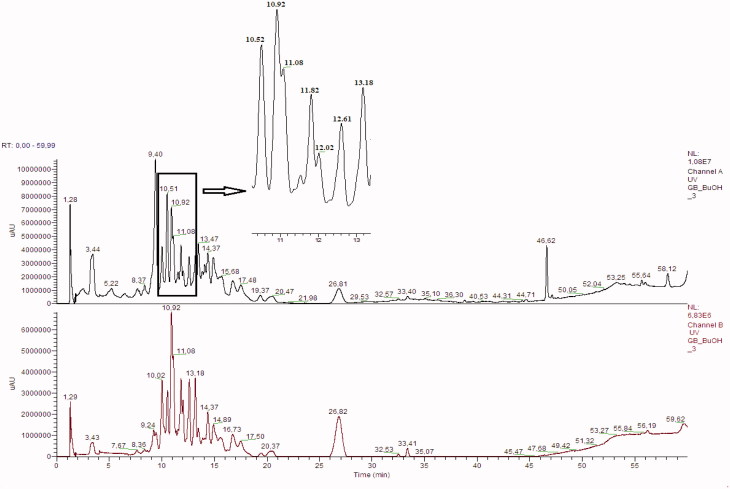
HPLC chromatogram of the butanol fraction of *L. barbarum* at 278 nm and 340 nm, respectively.

Seventeen different phenolic compounds were traced in our samples and their identification was achieved by studying their retention times, UV–Vis absorption spectrum in correlation to proposed MS fragmentation mechanisms and comparison to the scientific literature (Fang et al. 2002; Sakakibara et al. 2003). Typical HPLC–DAD–MS chromatograms are presented in Figures 3 and 4.

It has been observed, upon the comparison of LC-DAD-MS (ESI+) chromatogram data of methanol extract fractions (ethyl acetate and butanol fractions), that different compounds were present in these fractions (Tables 2 and 3). However, besides this difference in their content, some compounds were seen in both the fractions.

**Table 2.** Retention times (Rt), molecular ions ([M + H]^ +^), sodium adducts ([M + Na]^ +^), fragment ions (20 eV and 80 eV), UV absorptions (*λ*_max_), and tentative identification of phenolic compounds in ethyl acetate fraction of the fruit of *Lycium barbarum*.

*N*°	Rt (min)	[M + H]^+^	[M + Na]^+^	Fragment ion (*m/z*) at 20 eV	Fragment ion (*m/z*) at 80 eV	UV *λ*_max_ (nm)	Identification
1	3.43	197	219	153, 219	153, 197, 219	292	Dihydroisoferulic acid derivative
2	9.90	339	/	163, 192, 267, 311, 339	163, 192, 267, 311, 339	292, 296sh	Coumaroyl-quinic acid
3	11.50	355	377	163, 355, 377	163, 355	246, 328, 292sh	Chlorogenic acid
4	12.35	489	511	194, 489, 511	164	296, 250sh	Coumaroyl-, caffeoyl-caffeic acid
5	14.65	485	/	147	147, 165, 177, 339, 485	240, 308	Coumaroyl-, isoferuloyl-caffeic acid/Coumaroyl-, ferruloyl-caffeic
6	16.55	369	391	177, 195, 369	149, 177, 391	246, 318, 328sh	Feruloylquinic acid ester
7	19.17	517	/	166, 195	166, 195, 369, 517	296	Isoferuloyl-, dihydrocoumaroyl- quinic acid
8	27.48	611	633	303, 465, 611, 633	303, 465, 611, 633	256, 356, 322sh	Quercetin 3-*O*- hexose coumaric ester
9	32.41	609	649	149, 177, 314, 609, 627, 649	149, 177, 314, 368, 473, 627, 649	242, 292sh, 316	Isoferuloyl-, benzoyl-, protocatechoyl-quinic acid
10	38.14	653	675	165, 197, 625, 653	165, 197, 625, 675	242, 288sh, 318	Dihydroisoferuloyl-, coumaroyl-, protocatechoyl-quinic acid
11	39.43	653	675	165, 197, 625, 653	165, 197, 625, 675	242, 286sh, 292, 316	Isomer of the compound (no.10) with the acids in different positions

**Table 3.** Retention times (Rt), molecular ions ([M + H]^ +^), sodium adducts ([M + Na]^ +^), fragment ions (20 eV and 80 eV), UV absorptions (*λ*_max_), and tentative identification of phenolic compounds in butanol fraction of the fruit of *Lycium barbarum*.

*N*°	Rt (min)	[M + H]^+^	[M + Na]^+^	Fragment ion (*m/z*) at 20 eV	Fragment ion (*m/z*) at 80 eV	UV *λ*_max_ (nm)	Identification
12	9.40	/	/	/	165	240, 292	Coumaric acid derivative
13	10.02	529	/	163, 511, 529	147, 163, 177, 511, 529	240, 314	Coumaroyl-, dimethylcaffeoyl-quinic acid
14	10.51	529	/	163, 511, 529	147, 163, 177, 511, 529	292, 296sh	Isomer of the compound (no.13)
15	10.92	355	377	163, 355, 377	163, 355	285sh, 316, 328sh,	Chlorogenic acid
16	11.82	339	/	265, 311, 339	265, 311, 339	240, 314	Coumaroyl-quinic acid derivative
17	12.61	339	/	163, 265, 311, 339	163, 265, 311, 339	242, 320	Coumaroyl-quinic acid derivative
18	13.18	773	/	465, 773	303, 465, 627, 773	256, 356, 322sh	Quercetin 3-*O*-hexose-*O*-hexose-*O*-rhamnose
19	26.81	611	633	303, 465, 611, 633	303, 465, 611, 633	256, 356, 322sh	Quercetin 3-*O*- hexose coumaric ester

The compound eluted at Rt 3.43 min was suggested to be a derivative of a dihydroisoferulic acid (no.1). It showed [M + H]^ +^ at *m/z* 197, [M + Na]^ +^ at *m/z* 219 and a fragment ion at *m/z* 153, which corresponds to the loss of the CO_2_ group from the carboxylic acid function (Sánchez-Rabaneda et al. 2004). The peak at 9.40 min was detected to be coumaric acid derivative (no. 12) as its absorption maxima is 292 nm, which is typical for coumaric acid (Sakakibara et al. 2003).

At Rt 12.35 min a coumaroyl-, caffeoyl-caffeic acid (no.4) was detected. It yielded a pseudomolecular ion [M + H]^ +^ at *m/z* 489, a sodium adduct ion [M + Na]^ + ^ at *m/z* 511 and a fragment ion at *m/z* 194 [caffeic acid + CH_2_]^ +^ and *m/z* 164, which corresponds to the molecular weight of coumaric acid.

The ethyl acetate fraction contains tentatively identified coumaroyl-, isoferuloyl-caffeic acid/coumaroyl-, feruloyl-caffeic acid (no.5) at Rt 14.65 min with [M + H]^ + ^485. Fragments at *m/z* 339 are due to the loss of the coumaroyl unit, *m/z* 177 [ferulic/iso ferulic acid – OH]^ +^, *m/z* 147 [coumaric acid – OH]^ +^ and *m/z* 165 [caffeic acid – OH]^ +^.

**Figure 5.**
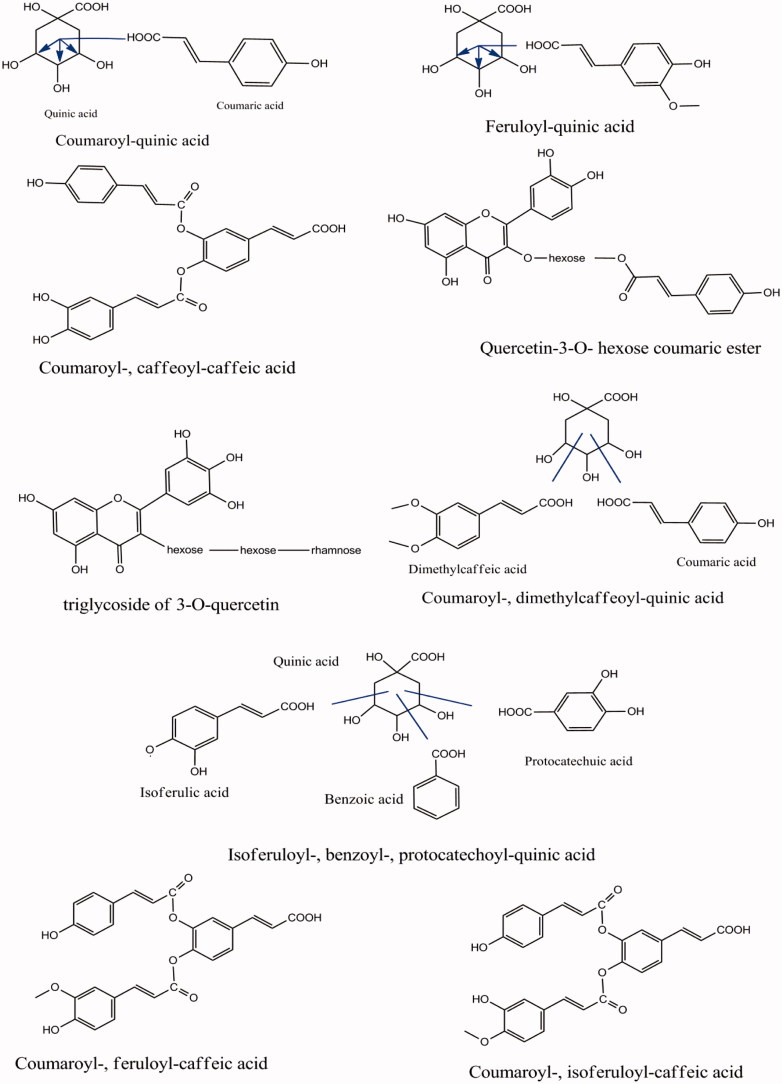
Proposed chemical structure of some compounds identified by the LC-DAD/MS of *L. barbarum* fractions.

Esters of hydrocinnamic and dihydroxybenzoic acids with quinic acids were dominant in our fractions (Figure 5). In most cases the quinic acid moiety does not give a fragment ion in ESI + analysis (Fang et al. 2002).

Ethyl acetate and butanol fractions have in their chromatograms three peaks at Rt 9.90, 11.82 and 12.61 min that correspond to a coumaroyl-quinic acid (no. 2) and a coumaroyl-quinic acid derivative (no. 16 and no. 17) The UV spectra are similar to that of the aforementioned *p*-coumaric acid in the literature (max 310 nm) (Clifford et al. 2006). MS showed a protonated molecular ion at *m/z* 339 compatible with a *p*-coumaroylquinic acid, with fragment ions at *m/z* 163 originating from the coumaric acid moiety, *m/z* 192 which was attributed to quinic acid, m/z 267 [M – (CH = CH – COOH)]^ +^, and *m/z* 311 [M – CO + H]^ +^. Derivatives of p-coumaric acid have been reported to be present in *Lycium* genus (Inbaraj et al. 2010; Abdennacer et al. 2015).

The butanol fraction contained a coumaroyl-, dimethylcaffeoyl-quinic acid (no. 13) at Rt 10.02 min, where [M + H]^ +^ is 529. The fragmentation pattern of the proposed compound showed a fragment ion at *m/z* 511 that results from the loss of water. Coumaric acid gave a typical fragment at *m/z* 147, while the fragment ion at *m/z* 163 is [dimethylcaffeic acid – COOH]^ +^. Loss of the methoxy group from the dimethylcaffeoyl unit (*m/z* 177) is observed as well.

The peak at Rt 10.92 min, revealed in the butanol fraction, and at Rt 11.50 min, revealed in the ethyl acetate fraction, shared the same UV spectra (λ_max_ 246, 316 nm, shoulder at 292 nm), although the MS spectra showed a pseudomolecular ion [M + H]^ +^ at *m/z* 355 corresponding to chlorogenic acid (no. 3 and 15). The fragmentation pattern of this assumed compound showed the fragment ions with the strongest and most distinguishable peaks at *m/z* 163 and 377, corresponding to the caffeoyl moiety and [M + Na]^ +^, respectively.

The UV and MS characteristics show feruloylquinic acid ester (no. 6) (Fang et al. 2002) in the ethyl acetate fraction at Rt 16.55 min. [M + H]^ +^ is 369 and [M + Na]^ + ^391. Fragments at m/z 195 correspond to the ferulic acid molecular ion, *m/z* 177 [ferulic acid – OH]^ +^ and *m/z* 149 [ferulic acid – COOH]^ +^.

The mass spectral analysis of the compound with Rt =19.17 min (ethyl acetate fraction) associated with the UV–vis spectrum (*λ*_max_ 296) identified a protonated molecular ion at *m/z* 517 that was attributed to isoferuloyl-, dihydrocoumaroyl- quinic acid (no. 7). The fragmentation pattern suggested that the fragment ion at *m/z* 369 corresponded to the loss of the dihydrocoumaroyl moiety. The ion at *m/z* 195 comes from the isoferulic molecular ion and *m/z* 166 corresponds to the molecular weight of the dihydrocouamric acid.

In the same fraction, the compound eluting at Rt 32.41 min was tentatively assigned the structure of isoferuloyl-, benzoyl-, protocatechoyl-quinic acid (no. 9). It yielded a pseudomolecular ion [M + H]^ +^ at *m/z* 609, a sodium adduct ion [M + Na]^ +^ at m/z 649 and fragment ions at *m/z* 627 [M + H_2_O + H]^ +^, *m/z* 473 [609 – protocatechoyl moiety]^ +^, *m/z* 368 [609 – protocatechoyl moiety – benzoyl moiety]^ +^, *m/z* 314 [368 – 54]^ + ^(54 fragments from quinic acid), *m/z* 177 [isoferulic acid − OH]^ +^ and *m/z* 149 [isoferulic – COOH]^ +^.

The ethyl acetate fraction also contained dihydroisoferuloyl-, coumaroyl-, protocatechoyl-quinic acid (no. 10) eluted at Rt 38.14 min and was characterized by a molecular ion at *m/z* 653 and [M + Na]^ +^ at *m/z* 675. The fragmentation pattern showed fragment ions at *m/z* 625 [M – CO + H]^ +^, *m/z* 197 [dihydroisoferulic acid + H]^ +^ and *m/z* 165 [coumaric acid + H]^ +^. The UV spectrum and the mass spectrum for the peak eluting at Rt 39.43 min suggested an isomer (no. 11) of the above-mentioned compound (no.16) with the acids in different positions.

The compound in the butanol fraction eluted at Rt 13.18 min was identified as a quercetin 3-*O*-hexose-*O*-hexose-*O*-rhamnose (no. 18) based on its pseudomolecular ion [M + H]^ +^ at *m/z* 773 with three noticeable fragments at *m/z* 627 = [(M + H) – rhamnosyl moiety]^ +^, *m/z* 465 [(M + H) – rhamnosyl moiety – hexosyl moiety]^ +^ and *m/z* 303 for the aglycon.

Rutin and quercetin 3*-O*-hexose coumaric ester present the same mass and the same first parent ion (*m/z* = 611) but were distinguished by comparing the UV absorption, the Rt and the MS fragmentation. The presence of the coumaroyl moiety bonded to the hexose retained the molecule longer in the reverse phase HPLC column compared to rutin, so we conclude that the molecule eluted at Rt 27.48 and 26.81 min in both the ethyl acetate and butanol fractions is quercetin 3-*O*-hexose coumaric ester (no. 8 and 19). It exhibited an intense molecular ion [M + H]^ +^ at *m/z* 611 and [M + Na]^ +^ at *m/z* 633. The ion at *m/z* 465 comes from the loss of the coumaroyl unit and the ion at *m/z* 303 corresponds to the aglycon.

## Conclusion

LC-DAD-MS (ESI+) profiling led to the identification of esters of hydrocinnamic and dihydroxybenzoic acids with quinic acids as the main antioxidant components. Among the flavonoids identified in this study, the presence of quercetin 3*-O*-hexose coumaric ester and quercetin 3-*O*-hexose-*O*-hexose-*O*-rhamnose, is reported for the first time in *Lycium barbarum*. Phenolic acids and their derivatives were among the compounds detected in this study. Coumaric, isoferulic, and caffeic acids, and their derivatives, were found to be the dominant ones. The presence of these compounds in *L. barbarum* may explain some of the health benefits observed in its traditional applications. Further investigations are needed to isolate and confirm the structural identity of the whole range of compounds detected in this study. It is worth noting that several compounds remained unidentified. Concerning the antioxidant activity, the ethyl acetate fraction was shown to have the best antioxidant activity, which can be related to the total content of phenolic compounds and flavonoids in this fraction. To conclude, these results suggest that consumption of goji berry fruits could serve as a good source of natural antioxidant compounds and that goji berry phenolic extracts could potentially be exploited for nutritional pharmaceutical purposes.

## References

[CIT0001] Abdennacer B, Karim M, Yassine M, Nesrine R, Mouna D, Mohamed B. 2015 Determination of phytochemicals and antioxidant activity of methanol extracts obtained from the fruit and leaves of Tunisian *Lycium intricatum* Boiss. Food Chem. 174:577–584. 25529722 10.1016/j.foodchem.2014.11.114

[CIT0002] Arnous A, Makris DP, Kefalas P. 2002 Correlation of pigment and flavanol content with antioxidant properties in selected aged regional wines from Greece. J Food Compos Anal. 15:655–665.

[CIT0003] Benzie IF, Strain JJ. 1999 Ferric reducing/antioxidant power assay: direct measure of total antioxidant activity of biological fluids and modified version for simultaneous measurement of total antioxidant power and ascorbic acid concentration. Meth Enzymol. 299:15–27. 9916193 10.1016/s0076-6879(99)99005-5

[CIT0004] Bucheli P, Vidal K, Shen L, Gu Z, Zhang C, Miller LE, Wang J. 2011 Goji berry effects on macular characteristics and plasma antioxidant levels. Optom Vis Sci. 88:257–262. 21169874 10.1097/OPX.0b013e318205a18f

[CIT0005] Carnés J, De Larramendi CH, Ferrer A, Huertas AJ, López-Matas MA, Pagán JA, Navarro LA, Garcia-Abujeta JL, Vicario S, Peña M 2013 Recently introduced foods as new allergenic sources: Sensitisation to Goji berries (*Lycium barbarum*). Food Chem. 137:130–135. 23200000 10.1016/j.foodchem.2012.10.005

[CIT0006] Clifford MN, Marks S, Knight S, Kuhnert N. 2006 Characterization by LC-MS^n^ of four new classes of *p*-coumaric acid-containing diacyl chlorogenic acids in green coffee beans. J Agric Food Chem. 54:4095–4101. 16756331 10.1021/jf060536p

[CIT0007] Endes Z, Uslu N, Özcan MM, Er F. 2015 Physico-chemical properties, fatty acid composition and mineral contents of goji berry (*Lycium barbarum* L.) fruit. J Agroaliment Proc Technol. 21:36–40.

[CIT0008] Fang N, Yu S, Prior RL. 2002 LC/MS/MS characterization of phenolic constituents in dried plums. J Agric Food Chem. 50:3579–3585. 12033832 10.1021/jf0201327

[CIT0009] Frankel EN, Meyer AS. 2000 The problems of using one dimensional methods to evaluate multifunctional food and biological antioxidants. J Sci Food Agric. 80:1925–1941.

[CIT0010] Gan L, Zhang SH, Yang XL, Xu HB. 2004 Immunomodulation and antitumor activity by a polysaccharide-protein complex from *Lycium barbarum*. Int Immunopharmacol. 4:563–569. 15099534 10.1016/j.intimp.2004.01.023

[CIT0011] Inbaraj BS, Lu H, Kao TH, Chen BH. 2010 Simultaneous determination of phenolic acids and flavonoids in *Lycium barbarum* Linnaeus by HPLC-DAD-ESI-MS. J Pharm Biomed Anal. 51:549–556. 19819093 10.1016/j.jpba.2009.09.006

[CIT0012] Istrati D, Vizireanu C, Iordachescu G, Dima F, Garnai M. 2013 Physico-chemical characteristics and antioxidant activity of goji fruits jam and jelly during storage. AUDJG-Food Technol. 37:100–110.

[CIT0013] Lau BW-M, Lee JC-D, Li Y, Fung SM-Y, Sang Y-H, Shen J, et al 2012 Polysaccharides from wolfberry prevents corticosterone-induced inhibition of sexual behavior and increases neurogenesis. PLoS One. 7:e33374. 22523540 10.1371/journal.pone.0033374 PMC3327693

[CIT0014] Lo AC, Yang D. 2015 *Lycium barbarum*: Neuroprotective effects in ischemic stroke In: *Lycium barbarum* and human health. Amsterdam, The Netherlands: Springer Netherlands; p. 125–134.

[CIT0015] Parejo I, Codina C, Petrakis C, Kefalas P. 2000 . Evaluation of scavenging activity assessed by Co(II)/EDTA-induced luminol chemiluminescence and DPPH* (2,2-diphenyl-1-picrylhydrazyl) free radical assay. J Pharmacol Toxicol Methods. 44:507–512. 11395328 10.1016/s1056-8719(01)00110-1

[CIT0016] Pérez-Jiménez J, Arranz S, Tabernero M, Díaz-Rubio ME, Serrano J, Goñi I, Saura-Calixto F. 2008 Updated methodology to determine antioxidant capacity in plant foods, oils and beverages: extraction, measurement and expression of results. Food Res Int. 41:274–285.

[CIT0017] Roberfroid MB. 2002 Global view on functional foods: European perspectives. Br J Nutr. 88(Suppl 2):133–138. 12495454 10.1079/BJN2002677

[CIT0018] Sakakibara H, Honda Y, Nakagawa S, Ashida H, Kanazawa K. 2003 Simultaneous determination of all polyphenols in vegetables, fruits, and teas. J Agric Food Chem. 51:571–581. 12537425 10.1021/jf020926l

[CIT0019] Sánchez-Rabaneda F, Jáuregui O, Lamuela-Raventós RM, Viladomat F, Bastida J, Codina C. 2004 Qualitative analysis of phenolic compounds in apple pomace using liquid chromatography coupled to mass spectrometry in tandem mode. Rapid Commun Mass Spectrom. 18:553–563. 14978800 10.1002/rcm.1370

[CIT0020] Song MK, Salam NK, Roufogalis BD, Huang THW. 2011 *Lycium barbarum* (Goji Berry) extracts and its taurine component inhibit PPAR-γ-dependent gene transcription in human retinal pigment epithelial cells: possible implications for diabetic retinopathy treatment. Biochem Pharmaco. 82:1209–1218. 10.1016/j.bcp.2011.07.089 21820420

[CIT0021] Termentzi A, Kefalas P, Kokkalou E. 2006 Antioxidant activities of various extracts and fractions of *Sorbus domestica* fruits at different maturity stages. Food Chem. 98:599–608.

[CIT0022] Verschuren PM. 2002 Functional foods: scientific and global perspectives. Br J Nutr. 88(S2):S126–S130. 10.1079/bjn2002675 12501832

[CIT0023] Yao X, Peng Y, Xu LJ, Li L, Wu QL, Xiao PG. 2011 Phytochemical and biological studies of *Lycium* medicinal plants. Chem Biodivers. 8:976–1010. 21674776 10.1002/cbdv.201000018

[CIT0024] Zhang R, Kang K, Piao MJ, Kim KC, Kim AD, Chae S, Park JS, Youn UJ, Hyun JW. 2010 Cytoprotective effect of the fruits of *Lycium chinense* Miller against oxidative stress-induced hepatotoxicity. J Ethnopharmacol. 130:299–306. 20546868 10.1016/j.jep.2010.05.007

[CIT0025] Zhang X, Zhou W, Zhang Y. 2015 Immunoregulation and *Lycium Barbarum* In: Lycium barbarum and human health. Amsterdam, The Netherlands: Springer Netherlands; p. 27–45.

